# Psychological telephone triage system for outpatient memory clinics - a way for adaptation to new challenges of increasing dementia prevalence and new treatment options?

**DOI:** 10.1016/j.ijchp.2024.100530

**Published:** 2024-12-06

**Authors:** Michaela Defrancesco, Fabienne Post, Alex Hofer, Juliane Jehle

**Affiliations:** University Hospital of Psychiatry I, Department of Psychiatry, Psychotherapy, Psychosomatics and Medical Psychology, Medical University of Innsbruck, Austria

**Keywords:** Dementia, Psychological intervention, Telephone triage system, Memory clinic

## Abstract

**Background:**

The increasing prevalence of dementia and new therapeutic developments for Alzheimer's disease (AD) have created an urgent need for rapid and cost-effective methods to diagnose those affected in the early stages of the disease. Unlike emergency departments, memory clinics lack triage systems, e.g. the Manchester Triage System.

**Method:**

This retrospective, observational study evaluated the effects of a psychological telephone triage (PTT) system for people requesting an initial assessment at a specialized outpatient memory clinic over a 15-months period in terms of waiting times, staff resources, and as a screening method for cognitive disorders. The PTT consisted of an interdisciplinary pre-screening of available preliminary patient information prior to telephone contact, a semi-structured interview of approximately 30 min with a clinical psychologist, and telephone psychological counseling if there was no indication for an on-site dementia assessment. Based on the PTT interview, patients were triaged using a 4-level priority system (red = acute, yellow = subacute, green = not acute, blue = no indication/counseling). The results were compared with data from the two years prior to the introduction of PTT.

**Results:**

The data of 612 people (327 before and 285 after the introduction of PTT) who called the secretary's office between January 1, 2021 and April 30, 2024 and requested an initial assessment were analyzed. Of the original sample who called after the introduction of PTT, 66.7% had an indication for an on-site visit and were invited to do so. This was accepted by 51.6%. A further 14% received psychological telephone counseling, resulting in a 34% reduction in on-site visits. Patients triaged as acute cases had the shortest waiting time and presented with the most severe cognitive and functional symptoms at the on-site visit.

**Discussion:**

Our study shows that PTT is an effective method to identify patients with urgent need for an initial dementia assessment and to provide psychological counseling as an alternative to on-site visits. We expect that this will reduce the number of emergency admissions and thus the burden on caregivers and the healthcare system. This PTT concept can thus help to better manage the increasing need for initial assessments in the context of new therapies for AD and the increasing prevalence of dementia in general.

## Introduction

In parallel with an aging population, the prevalence of dementia and the associated economic burden are increasing dramatically, creating an urgent need for rapid and cost-effective methods to diagnose and monitor people with neurocognitive disorders in the early stages of the disease. The expansion of diagnostic options is therefore highly relevant to health policy in order to improve patient outcomes and enable timely intervention.

An estimated 50 million people worldwide have dementia and this number is expected to reach 152.8 million by 2050 (Collaborators, 2022). Given this significant increase in the number of older people with cognitive impairment or dementia and the emergence of new treatment options, the need for specialized health care services for dementia screening will increase. Alzheimer´s disease (AD) is the most common cause of dementia accounting for 60% to 80% of cases. The understanding of AD has undergone significant evolution in recent years. There is growing evidence supporting the existence of an extended preclinical phase characterized by minimal clinical or cognitive impairment but detectable neurometabolic and microstructural alterations in the brain Sperling et al. (2011). Following the preclinical phase, patients develop mild cognitive impairment (MCI) and ultimately progress to the clinical manifestation of AD (Petersen, 2004; Sperling et al., 2011). MCI is common in older people and can be a transitional stage between normal aging an dementia (Petersen, 2004). However, in approximately 50% of patients, the etiology associated with MCI is not related to neurodegenerative disease and has reversible causes such as psychiatric disorders (e.g., major depression) or comorbid medical conditions (Burns & Zaudig, 2002). The differential diagnosis between MCI due to AD or other neurodegenerative diseases and MCI due to other etiologies is therefore challenging, and the number of patients continues to rise. In contrast, pharmacological treatment options for people with neurocognitive disorders are limited to symptomatic therapy. Recently, the U.S. Food and Drug Administration (FDA) has approved the monoclonal antibodies (mAB) aducanumab and lecanemab for the treatment of amyloid positive patients with MCI and early AD, and European approvement for lecanemab is expected in 2024 or 2025. In addition, a further mAB, donanemab, has also shown positive biological and clinical results in a Phase 3 clinical trial in patients with MCI and mild AD and has been submitted to the FDA and the European Medicines Agency (EMA) for approval (Sims et al., 2023).

Memory clinics play a crucial role in the early detection and intervention of dementia and other neurocognitive disorders, as well as providing ongoing care and support to improve the quality of life for those affected and their caregivers (Borson et al., 2013). These are outpatient clinics for selected patients with scheduled appointments, and the capacity for patient appointments is fixed and cannot be increased flexibly to meet demand. In contrast to emergency departments, triage systems such as the Manchester Triage System (MTS) are lacking for memory clinics. Although previous studies have investigated the suitability of brief cognitive assessments and remote assessments for classifying and selecting patients for in-depth assessment in specialized memory outpatient clinics (Beinhoff et al., 2005; Dautzenberg et al., 2022), as well as the suitability of telemedical support for dementia patients, validated triage protocols for memory outpatient clinics to optimize scheduling and offer suitable alternatives when dementia assessment is not indicated are still lacking. On the other hand, the expected significant increase in the number of people with dementia requires a considerable increase in staffing and structural resources for the assessment and treatment of people with suspected cognitive impairment, MCI, or dementia. In particular, the approval of mAB therapies for early clinical stages of AD will increase the need for clinical assessments of people with mild cognitive deficits. In the short-to-medium term, the psychological telephone triage (PTT) system presented in this paper may therefore help memory clinics to better select patients who are potentially eligible for new dementia treatments. We analyzed the efficacy, sensitivity, and applicability of this newly introduced method at our memory outpatient clinic over a period of 15 months and compared the waiting times for initial dementia assessment in different diagnostic groups with varying degrees of urgency before and after PTT introduction. In addition, we present the results of the information gathered during PTT and the on-site visit, as well as the impact of PTT on the time and human resources in our memory clinic.

## Material and methods

### Study design

This retrospective, observational study evaluated the effects of a psychological telephone triage system for patients requesting an initial assessment at our memory clinic on waiting times, indication clarification, and as a screening method for cognitive disorders between January 1, 2021 and April 30, 2024. Before initiation of PTT in February 2023, appointments for initial assessments were scheduled by a secretary. As of February 1, 2023, all persons requesting an appointment for initial dementia assessment were scheduled for PTT. Data were collected from people who called the secretary's office requesting an initial assessment at a psychiatric memory outpatient clinic.

### Background for the development of PTT

Our memory clinic is one of three specialized outpatient clinics for the assessment and treatment of patients with dementia and other neurocognitive disorders in a middle-European federal province with a population of 776,000, of which 13,000–14,000 currently suffer from dementia. In the three memory clinics (two psychiatric, one neurological), about 1700 patients per year from all over the province are initially assessed for memory deficits.

Dementia assessments can also be performed by psychiatrists or neurologists in private practice or in smaller hospitals. However, detailed biomarker diagnostics for amyloid and tau markers as well as high-resolution imaging with 3 Tesla MRI are only available in the three specialized memory clinics.

Due to a significant increase in the need for appointments at our memory clinic and the resulting increase in waiting times up to 9 months for an initial assessment, we developed the PTT service in early 2023. By April 30, 2024, the responsible psychologist had conducted 250 telephone triage interviews based on a newly developed PTT interview protocol.

### Procedure for making initial appointments before and after the introduction of PTT

Appointments at our outpatient memory clinic can be made daily by telephone via the secretary's office. Prior to the implementation of PTT, all appointments (initial assessments and check-ups) were scheduled by the secretary's office according to availability.

Since the initiation of PTT (February 1, 2023), all callers (patients, relatives, referrals) requesting an initial assessment receive a PTT telephone appointment (time slot with 30 min) with a clinical psychologist via the secretary's office within 1–3 working days. Existing patient information (from the secretary and medical history in the local and national electronic patient record) of the scheduled patient is reviewed prior to the PTT interview and discussed in the daily interdisciplinary memory clinic meeting with psychiatrists and psychologists.

Any available preliminary patient information (e.g., recent surgery, cerebral imaging findings, digitally recorded premedication, known mental retardation, hearing or visual impairment, migration background) relevant for the planning of the initial assessment is briefly discussed. This preliminary review of patient information and interdisciplinary discussion allows additional acute appointments to be scheduled for people who are more likely to require acute triage, after discussion of current staffing resources. Furthermore, the pre-screening of existing patient information is important to individually prepare PTT advice for the patient (such as providing information regarding regional care providers depending on the place of residence). It also helps to identify appointments at other memory clinics and hence avoid double bookings.

### Psychological telephone triage (PTT) interview

The PTT is a semi-structured interview lasting approximately 30 min, in which the pre-defined content of the documentation form is collected. At the beginning, the reason for the appointment, socio-demographic information and relevant previous findings (e.g., psychiatric or neurological pre-treatments) are asked. In the second part of the interview, cognitive, emotional, and behavioral symptoms and deficits in everyday functions over the past month are evaluated. The questions are derived from the six items of the Clinical Dementia Rating (CDR) scale (Morris, 1993). Finally, the current care situation is assessed, with a focus on the financial situation and the burden on family caregivers. We also ask whether there are any factors that make timely dementia assessment necessary (e.g., urgent need for institutionalization and/or legal representation). The detailed pre-screening protocol and the PTT survey documentation is presented in Appendix 1.

### Description of the 4-level PTT system

Following the PTT interview, the triage code is set based on a 4-level priority system. The levels are as follows: level 1 (green, not acute), level 2 (yellow, subacute), level 3 (red, acute), and level 4 (blue, no indication for on-site initial dementia assessment). Appointments are allocated based on the assigned triage code. The level is comprised of the following elements: the indication for dementia assessment, the questions based on the CDR interview, the care situation, the presence of behavioral and psychological symptoms in dementia (BPSD), general risk factors for dementia, and time and/or financial urgency.

Patients with a red triage receive an appointment within 1–3 weeks according to availability. Patients with a yellow triage receive an appointment within a period of 4–12 weeks, and patients with a green triage within a period of 3–4 months. Callers with a blue triage receive PTT telephone counseling. The detailed 4-level PTT system is presented in Appendix 2.

### PTT telephone counseling (blue triage)

Callers who have been identified as not requiring or not eligible for an on-site dementia assessment (blue triage) are provided with further psychological counseling based on their needs. This involves psychoeducation regarding dementia and other possible reasons of cognitive impairment. The counseling is designed to help strengthen resources and learn strategies to improve cognitive and emotional functioning. Information is also provided on who to contact or where to seek further support or treatment.

### On-site initial dementia assessment

Each patient and, if possible, a relative or caregiver is interviewed in a comprehensive manner at the initial on-site appointment. Information is obtained on somatic comorbidities as well as currently prescribed psychotropic and other medication and matched with PTT documentation. Patients are scheduled for cerebral imaging (magnetic resonance imaging [MRI] or computer tomography [CT]), and, if indicated, an amyloid positron emission tomography (PET) scan, a fluorodeoxyglucose (FDG) PET scan or lumbar puncture. Routine blood sampling is also performed.

### On-site neuropsychological assessment

During initial assessment, all patients complete a neuropsychological assessment including the Mini Mental State Examination (MMSE) (Folstein et al., 1975) and subtests of the “Consortium to Establish a Registry for Alzheimer's Disease” (CERAD) battery (Rosen et al., 1984). This battery includes subtests for verbal memory and recognition (word list learning, word list delayed recall, and word list recognition), constructional praxis (figure drawing), figural memory (delayed recall), confrontational object naming (Boston Naming Test [BNT] – short version), verbal fluency (animals/min, s-words/min), cognitive flexibility (Trail making Test A and B), and Clock Drawing (Royall et al., 1998).

### On-site scales and questionnaires

Symptoms from six domains (Memory, Orientation, Judgment and Problem Solving, Community, Home and Hobbies, and Personal Care) are assessed using the CDR assessment protocol. The answers are rated in the six domains according to their severity (0 not present, 0.5 questionable, 1 mild, 2 moderate, 3 severe). The CDR score (0–3) is calculated based on the published algorithm. Depressive symptoms are assessed using the 30-items version of the Geriatric Depression Scale (GDS) (Yesavage et al., 1982). The Caregiver Burden Interview (CBI) (Zarit et al., 1980), a self-rating questionnaire comprising 22 questions, is used to assess caregiver burden (CB) in family members at the time of initial assessment. The sum score of the 22 questions is classified according to the literature: 0–20 indicate absent or little CB, 21–40 mild to moderate CB, 41–60 moderate to severe CB, and 61–88 severe CB. Instrumental activities of daily living are rated on the Lawton Instrumental Activities of Daily Living Scale (IADL) (Lawton & Brody, 1969). Scores range from 0 (low function, dependent) to 8 (high function, independent). The Neuropsychiatric Inventory (NPI) (Cummings, 1997), is used to assess frequency (range: 0–4 points), severity (1–3 points), and emerging caregiver burden (0–5 points) of twelve behavioral and psychological symptoms. The total score ranges from 0 (no BPSDs) to 144 (severe BPSD).

### Diagnostic criteria

In clinical practice at our memory clinic, patients are classified as “cognitively intact” (CI) when they 1) report mild self-experienced cognitive decline compared to a previous normal state unrelated to an acute event or explained by significant psychiatric or somatic disease according to the criteria of subjective cognitive decline (SCD) (Jessen et al., 2014) or no self-experienced cognitive decline compared to a previous normal state, and 2) do not fall short of the threshold of 1 standard deviation (SD) below the mean of normative data derived from a representative sample in the neuropsychological test battery and a CDR score of 0. MCI is diagnosed according to the criteria of Petersen et al. (Petersen et al., 2001), i.e. in patients reporting subjective memory complaints over the previous 6 months and showing impaired memory function (verbal or figural) in the neuropsychological assessment >1.5 SD below the mean of normative data and additionally having a CDR score between 0 and 0.5. Dementia of any etiology (Alzheimer´s dementia, vascular dementia (VD), dementia due to Chorea Huntington, Parkinson´s disease, or Pick's disease (other dementia)) is diagnosed (ICD-10 criteria) in case of 1) presence of subjective memory complaints over the past 6 months, 2) impaired neuropsychological function >2 SD in one memory function (verbal or figural memory) and at least one other cognitive domain, 3) deficits in activities of daily living assessed with a clinical interview, and 4) a CDR score ≥1.

Cognitive impairment due to another etiology (e.g., due to major depression or other mental health disorder, substance abuse, normal pressure hydrocephalus) or cause is diagnosed according to ICD-10 criteria.

### Statistical analysis

For statistical analysis, study participants were assigned to the following diagnostic subcategories: CI, MCI, dementia (including dementia of any etiology), and others (including cognitive impairment due to another etiology). Statistical analysis was conducted with IBM SPSS (version 29). The significance level was set to α = 5%. The analytical focus was placed on a comparison of diagnostic subcategories and triage level regarding demographics, clinical characteristics, waiting time and examination results. These groups were compared by means of Kruskal-Wallis Test or Mann-Whitney-U test for metric variables and Chi-square test for categorical variables. Post-hoc comparisons were adjusted by Dunn-Bonferroni method. Additionally, we were interested in the association of waiting time following PTT and clinical presentation at on-site visit. Therefore, the Spearman correlation for metric variables was employed. Patient characteristics were summarized using descriptive statistics (mean, standard deviation (SD), count, percentage).

This retrospective study was approved by the Ethics Committee of the Medical University of Innsbruck, Austria (approval number 1046/2018). Due to the retrospective study design, patients were exempted from signing an informed consent.

## Results

**Fig. 1:**
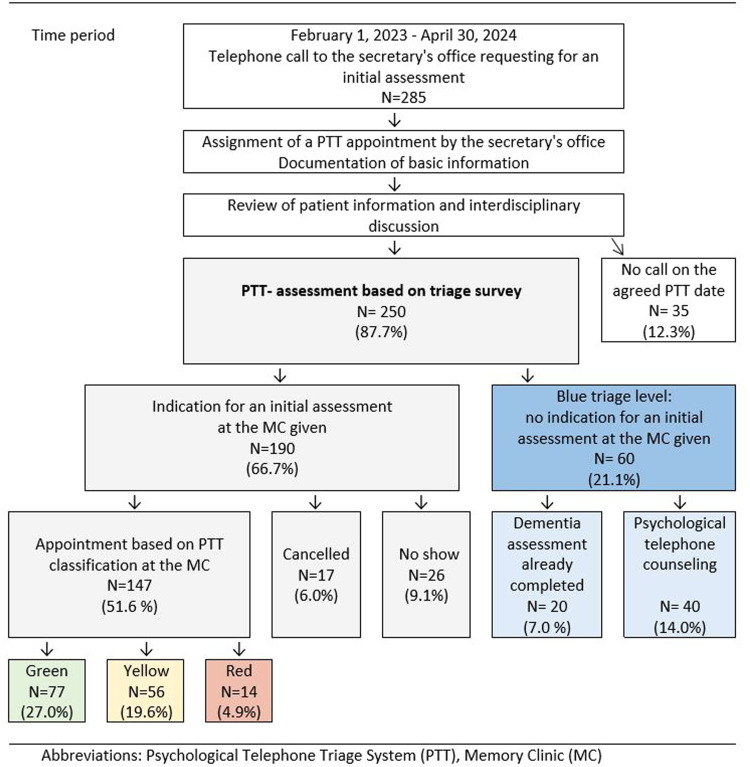
Study flow-diagram and results of Psychologic Telephone Triage between February 1st, 2023 and April 30th, 2024 caption under Fig. 1 Abbreviation: Memory Clinic (MC).

A total of 612 people (327 before and 285 after the introduction of PTT) called the secretary's office between January 1, 2021 and April 30, 2024 and requested an initial assessment either for themselves or for a relative to assess memory complaints. Out of those who called prior to PTT implementation, 295 (90.2%) came to their scheduled appointment. Of the 285 callers (39% patients, 61% caregivers) who received a PTT telephone appointment via the secretary's office, 147 (51.6%) came to the memory clinic for an initial assessment. 43 (15.1%) missed the scheduled appointment, 60 (21.1%) had no indication for an initial assessment but received psychological telephone counseling or further information, and 35 (12.3%) did not call for a PTT appointment. Details of the PTT study flow-diagram are presented in Fig. 1.

### Demographic and clinical characteristics before and after the introduction of PTT

Table 1 summarizes sociodemographic data and characteristics of patients before and after the introduction of PTT. Of all outpatients who presented prior to the introduction of PTT, 99 met the diagnostic criteria for MCI (mean age 72.12±10.56, 61.6% female), 127 (mean age 79.7 ± 6.9, 64.6% female) for dementia (69.5% AD, 18% VD, 12.5% other dementia), 30 had mild to major cognitive impairment due to other diseases (mean age 67.1 ± 10.2, 56.7 % female), and 39 had no significant cognitive impairment (mean age 69.7 ± 9.8, 56.4% female)

**Table 1:** Demographic and clinical characteristics of outpatients at initial assessment at a specialized memory clinic before and after the introduction of a psychological telephone triage system.

	Before PTT introduction *N* = 295	After PTT introduction *N* = 147	Test statistics^a^	df	p-value
	Mean ± SD or N/%				
Age (years)	75.54±10.23	73.20±13.03	*Z*= - 0.347	—	0.728
Sex (% female)	184 (61.5)	91 (61.9)	χ² = 0.246	1	0.350
Education (years)	10.47±3.01	10.09±3.07	*Z*= - 0.874	—	0.382
MMSE total score	23.67±5.49	23.32±5.10	*Z*= −1.128	—	0.259
NPI total score	11.01±8.03	11.90±11.71	*Z*= −2.097	—	0.036
CDR total score	0.71±0.53	0.84±0.56	*Z*= −0.862	—	0.389
IADL total score	6.14±2.05	6.23±1.32	*Z*= −0.762	—	0.235
Diagnostic category					
Cognitively intact	39 (13.2)	7 (4.8)	χ²= 15.883	3	<0.001
MCI	99 (33.6)	36 (24.5)			
Dementia	127 (43.1)	78 (53.1)			
Other	30 (10.2)	26 (17.7)			
Waiting time total (days)	71.7 ± 44.16	62.82±47.29	*Z*= −1.263	—	0.206
Waiting time (days) per diagnostic category					
Cognitively intact	43.00±52.21	82.41±55.33	*Z*= −1.652	—	0.099
MCI	63.45±41.27	71.08±50.58	*Z*= −1.274	—	0.203
Dementia	73.50±41.52	52.72±42.98	*Z*= −3.240	—	0.001
Other	70.60±43.6	87.00±44.70	*Z*= −1.750	—	0.080

Abbreviations: standard deviation (SD), Psychological Telephone Triage (PTT) System, Mild Cognitive Impairment (MCI), Mini Mental State Examination (MMSE), Neuropsychiatric Inventory (NPI), Clinical Dementia Rating Scale (CDR), Instrumental Activities of Daily Living Scale (IADL)

a Mann-Whitney *U* test was used for metric and Chi-square test for nominal variables

Of the outpatients who presented after PTT introduction, 36 met the diagnostic criteria for MCI (mean age 68.1 ± 13.3, 67.6% female), 78 (mean age 79.8 ± 8.9, 65.8% female) for dementia (69.5% AD, 18% VD, 12.5% other types of dementia), 26 had mild to major cognitive impairment due to other diseases (mean age 60.8 ± 12.4, 63.0% female; 84.6% major depression, 7.7% bipolar disorder, 7.7% alcohol-related dementia), and 7 had no significant cognitive impairment (mean age 72.6 ± 6.7, 57.1% female). The distribution of diagnoses differed significantly (χ² (6) = 32.227, *p* < 0.001, φ = 0.47) depending on the three triage levels (green, yellow, red). The prevalence of MCI diagnosis was highest in the green triage group, whereas the prevalence of dementia diagnoses was highest in the yellow and red triage groups, with an increase from the green to the red triage level.

### Demographic and clinical characteristics at initial on-site assessment based on ptt classification

Based on the 4-level PTT system, the majority of patients who came for initial assessment were classified as green (52.4%), followed by yellow (38.1%) and a minority as red (9.5%). The three groups differed significantly in terms of age, global and cognitive functioning (MMSE score, CDR score), activities of daily living (IADL score), behavioral and psychological symptoms of dementia (NPI total score), and caregiver burden (CBI total score). The majority of patients who were assigned to the green triage level were married (55%) and did not require nursing care (74%). In contrast, most patients with a red triage level lived alone (57%) and required care and support from their family (43%).

Analysis of waiting time for initial assessment showed that the green triage group had the longest waiting time, followed by the yellow and red groups. A post hoc group comparison revealed no significant differences between the yellow and red groups for any of the measures, with the exception of the NPI score, which was higher in the red group. Details are shown in Table 2, Table 3, Table 4.

**Table 2:** Demographic and clinical characteristics of outpatients at initial assessment at a specialized memory clinic following categorization according to PTT classification.

	PTT classification						Post-hoc-test^b^		
	Green *N* = 77	Yellow *N* = 56	Red *N* = 14	Test statistics^a^	df	*p*-value	Green vs. Yellow	Green vs. Red	Red vs. Yellow
	Mean ± SD, N (%)								
Age (years)	68.52 ± 13.51	77.02 ± 10.95	83.71 ± 4.86	*H=26.488*	2	< 0.001	< 0.001	< 0.001	0.105
Education (years)	10.35 ± 3.16	9.98 ± 2.61	9.15 ± 4.20	*H=3.542*	2	0.170	0.982	0.213	0.697
Sex (% female)	51 (62.2)	38 (67.9)	8 (57.1)	*χ² =0.577*	2	0.749	—	—	—
NPI total score	10.25 ± 10.43	11.64 ± 12.32	21.08 ± 12.11	*H=10.078*	2	0.006	1.000	0.005	0.021
MMSE total score	25.05 ± 3.62	22.02 ± 5.34	19.08 ± 7.17	*H=17.797*	2	< 0.001	0.002	0.003	0.573
CDR total score	0.59 ± 0.34	1.04 ± 0.55	1.38 ± 0.74	*H=31.796*	2	< 0.001	< 0.001	< 0.001	0.685
IADL total score	6.92 ± 1.47	5.50 ± 1.67	4.43 ± 1.99	*H=33.937*	2	< 0.001	< 0.001	< 0.001	0.319
CBI total score	14.97 ± 12.32	27.59 ± 14.22	37.75 22.87	*H=13.995*	2	< 0.001	0.002	0.055	1.000
Waiting time (days)	77.40 ± 47.73	53.37 ± 41.11	20.36 ± 32.56	*H=17.810*	2	< 0.001	0.015	< 0.001	0.131

Abbreviations: standard deviation (SD), Psychological Telephone Triage (PPT) System, Mini Mental State Examination (MMSE), Neuropsychiatric Inventory (NPI), Clinical Dementia Rating Scale (CDR), Instrumental Activities of Daily Living Scale (IADL), Caregiver Burden Interview (CBI).

a Kruskal-Wallis Test was used for metric and Chi-square test for nominal variables.

b Dunn-Bonferroni-Test corrected for multiple comparison.

**Table 3:** Differences in the waiting time for an initial assessment in a specialized memory clinic after PTT classification and by diagnosis.

	PTT classification						Post-hoc-test^b^		
Waiting time (days)	Green *N* = 77	Yellow *N* = 56	Red *N* = 14	Test statistics^a^	df	*p*-value	Green vs. Yellow	Green vs. Red	Red vs. Yellow
	Mean ± SD N (%)								
Cognitively intact	42.80 ± 57.52 *N* = 5 (6.5%)	43.50 ± 55.86 *N* = 2 (3.6%)	— *N* = 0 (0%)	*Z* = −0.391	—	0.857	—	—	—
MCI	81.41 ± 48.33 *N* = 29 (37.7%)	32.33 ± 38.80 *N* = 6 (10.7%)	4.0 ± 0.0 *N* = 1 (7.1%)	*Z* = - 2.100	–	0.036	—	—	—
Dementia	71.96 ± 49.18 *N* = 24 (31.2%)	50.21 ± 36.18 *N* = 42 (75.0%)	23.00 ± 34.64 *N* = 12(85.7%)	*H=9.785*	2	0.008	0.255	0.006	0.132
Other	87.26 ± 40.72 *N* = 19 (24.7%)	99.83 ± 49.80 *N* = 6 (10.7%)	5.0 ± 0.0 *N* = 1 (7.1%)	*Z* = −0.924	–	0.366	—	—	—

Abbreviations: standard deviation (SD), Psychological Telephone Triage (PTT) System, Mild Cognitive Impairment (MCI).

a Kruskal-Wallis Test or Mann-Whitney-U test was used for metric variables.

b Dunn-Bonferroni-Test corrected for multiple comparison.

**Table 4:** Sociodemographic characteristics of people with initial assessment at a specialized memory clinic following categorization according to PTT criteria.

		PTT classification					
Sociodemographic variables	Total *N* = 147	Green *N* = 77	Yellow *N* = 56	Red *N* = 14	Test statistics^a^	df	*p*-value
	N (%)						
Living situation							
Alone	59 (40.1)	24 (31.2)	27 (48.2)	8 (57.14)	*χ² = 7.727*	4	0.102
With partner/family	87 (59.2)	53 (68.8)	28 (50.0)	6 (42.86)			
Institutionalized	1 (0.7)	0	1 (1.8)	0			
Marital status							
Single	16 (10.9)	9 (11.7)	7 (12.5)	0	χ² = *13.486*	6	0.036
Married	71 (48.3)	42 (54.6)	23 (41.1)	6 (42.86)			
Divorced/Separated	22 (15.0)	13 (16.9)	9 (16.1)	0			
Widowed	38 (25.9)	13 (16.9)	17 (30.4)	8 (57.14)			
Care situation							
None	91 (61.9)	57 (74.0)	29 (51.8)	5 (35.71)	*χ² = 14.782*	6	0.022
Outpatient/day care	25 (17.0)	8 (10.4)	14 (25.0)	3 (21.43)			
24-hour nursing care	3 (2.0)	1 (1.3)	2 (3.6)	0			
Family care	28 (19.1)	11 (14.3)	11 (19.6)	6 (42.86)			

Abbreviation: Psychological Telephone Triage (PPT) System.

a Chi-square test for nominal variables.

Table 5 shows the correlations between the waiting times and the clinical findings recorded during the on-site visit. We found a significant association between shorter waiting times and older age, greater caregiver burden (CBI total score), and greater deficits in cognition (MMSE total score) and activities of daily living (IADL score).

**Table 5:** Correlation between waiting time and clinical and demographic variables after PTT implementation.

	Spearman correlation							
Variable		1	2	3	4	5	6	7
1. Waiting time (days)	n	147						
2. Age (years)	*Spearman r*	−0.256	–					
	*p*-value	0.002	.					
3. MMSE total score	*Spearman r*	0.092	−0.352	–				
	*p*-value	0.278	<0.001	.				
4. CDR total score	*Spearman r*	−0.149	0.561	−0.667	–			
	*p*-value	0.098	<0.001	<0.001	.			
5. NPI total score	*Spearman r*	−0.061	−0.032	−0.148	0.144	–		
	*p*-value	0.501	0.726	0.099	0.109	.		
6. IADL total score	*Spearman r*	0.188	−0.491	0.619	−0.825	−0.220	–	
	*p*-value	0.024	<0.001	<0.001	<0.001	0.014	.	
7.CBI total score	*Spearman r*	−0.296	0.398	−0.463	0.535	0.173	−0.565	–
	*p*-value	0.017	0.001	<0.001	<0.001	0.199	<0.001	.

Abbreviations: Psychological Telephone Triage (PTT) system, Mini Mental State Examination (MMSE), Neuropsychiatric Inventory (NPI), Clinical Dementia Rating Scale (CDR), Instrumental Activities of Daily Living Scale (IADL), Caregiver Burden Interview (CBI).

### Information recorded as part of the PTT and assignment to a specific triage level

**Fig. 2:**
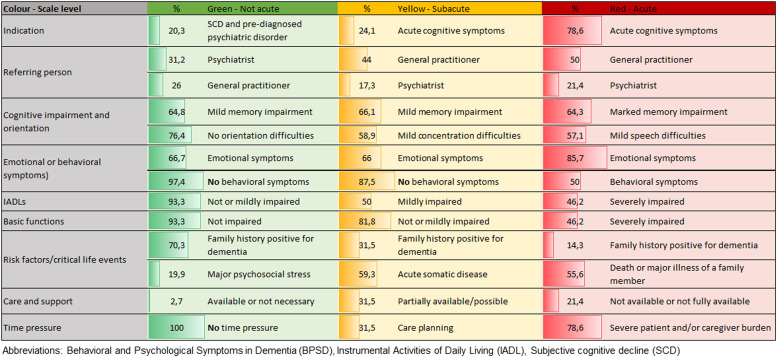
Analysis of most prevalent symptoms and factors in the three levels assessed by Psychological Telephone Triage (PTT) of patients with indication for on-site dementia assessment caption under the Fig. 2 Abbreviations: Behavioral and Psychological Symptoms in Dementia (BPSD), Instrumental Activities of Daily Living (IADL), Subjective cognitive decline (SCD).

68% of PTT calls were made by the patients themselves. People who were assigned to the blue triage level and received psychological counseling (*N* = 40, 60% female) were younger (age 62.0 ± 17.0, range 22–86 years) than those assigned to one of the other three triage levels. 68% of the people invited to an on-site visit were undergoing psychiatric, neurological, or psychotherapeutic treatment, 65% had a positive family history for dementia, and 85% reported a recent critical life event that was in close association with subjective cognitive decline. Fig. 2 provides an overview of the most common criteria that led to classification into a particular triage level and were associated with an invitation to an on-site dementia assessment.

### Impact of PTT on the waiting time for an initial assessment of memory complaints

Prior to the implementation of PTT, the waiting time for an initial assessment at the memory clinic did not correlate significantly with patients’ age (r²= −0.094, *p* = 0.104), the MMSE score (r²= 0.002, *p* = 0.973), the NPI total score (r²= −0.060, *p* = 0.344), the CDR total score (r²= 0.034, *p* = 0.597), or the IADL total score (r²= −0.005, *p* = 0.931).

Over time, there was a significant increase in waiting times for an initial assessment between the years 2021 (49.5 ± 30.5 days), 2022 (70.01±33.9 days) and until January 31, 2023 (135.0 ± 45.2 days) (Kruskal-Wallis test: *H* = 92.812, df = 2, *p* = <0.000). Patients who made an appointment for a first on-site visit through the secretary's office on January 31, 2023 had a waiting time of 273 days. The maximum waiting time after the introduction of PTT on February 1, 2023 was 105 days.

After the implementation of PTT, the total waiting time decreased slightly, although not significantly, by an average of 9 days. A significant difference was observed in the waiting times for patients with MCI and dementia. The red triage group had the shortest waiting times, followed by the yellow and green groups. Detailed results are presented in Table 3.

### Impact of PTT on time and staff resources

Before implementation of PTT, 90.2% of people (*N* = 295) scheduled for an initial consultation by the secretary's office attended the on-site visit. The time required for an initial assessment is approximately three and a half hours per patient, encompassing a clinical examination, neuropsychological examination, discussion of findings, and documentation. After the introduction of PTT, the number of on-site appointments decreased to 66.7% (*N* = 190) of the initial applications for a dementia assessment submitted via the secretary´s office (*N* = 285). Approximately 27% of callers did not call for PTT triage or canceled or missed their scheduled on-site appointment, and another 21% needed a telephone consultation or had no indication for an initial on-site assessment. It can be assumed that without PTT in the period from February 1, 2023 to April 30, 2024, 90% of people who would have made an appointment via the secretary's office would have attended an on-site consultation (approximately 257 patients, time spent 900 h). The implementation of PTT led to a 33% reduction in on-site visits, resulting in a 22% reduction in total time spent (197 h) despite the additional time spent on PTT (45 min per patient).

## Discussion

This study assessed the effect of a newly developed psychological telephone triage system for people requesting an initial dementia assessment at a specialized outpatient memory clinic. As an important finding, we were able to show that telephone triage by an experienced psychologist can significantly save time and staffing resources and leads to a reduction of waiting time depending on patients’ and caregivers’ needs. Over the course of 15 months of PTT, we were able to reduce the number of on-site visits by 33% through psychological telephone counseling and interdisciplinary pre-screening of those seeking help. This made it possible to use limited resources in a more needs-oriented manner and in line with individual requirements. In addition, the introduction of PTT reduced the maximum waiting time for patients from 9 months to an average of 3.5 months.

Although telephone triage cannot replace a detailed assessment of cognitive impairment, the symptoms recorded in the PTT interview were largely consistent with those recorded during the on-site visit. We also received feedback that individuals with cognitive deficits or dementia and their families perceived the PTT service and the opportunity to discuss their concerns with a psychologist immediately as a great relief. As the approval of new therapies for Alzheimer's disease is expected to lead to a considerable increase in the need for dementia assessments, the establishment of resource-efficient methods such as PTT, which can be used to screen individuals with suspected dementia, is of utmost importance in terms of health economics.

A comparison of our results with those of previous studies is only possible to a limited extent, as no triage protocols for outpatient memory clinics have yet been published. In fact, most studies using telephone triage focused on older patients and/or patients with dementia and assessed the need for an emergency department visit (Inokuchi et al., 2021; Savioli et al., 2024) or dealt with the effectiveness of telephone-based support for dementia patients and their caregivers. The Care Ecosystem Randomized Clinical Trial (Possin et al., 2019), for example, investigated collaborative dementia care delivered via telephone and internet channels with the aim of improving quality of life, caregiver well-being, and health care utilization among people with dementia. The authors found positive effects of the telephone intervention on the quality of life and stress of patients and caregivers, which is in line with our experience with telephone psychological counseling so far.

As mentioned previously, in contrast to our PTT system, the telephone intervention studies published to date have focused primarily on telephone support for patients with dementia, caregivers, or the general elderly population (Mavandadi et al., 2017; Waller et al., 2017) and did not specifically use this option to clarify the need for an on-site dementia assessment.

Although the field of telemedicine in geriatrics has become increasingly important (Haimi & Sergienko, 2024; Murphy et al., 2020; Nkodo et al., 2022) and remote dementia assessments have now been developed and evaluated, telemedicine has not been shown to be sufficiently convincing as a stand-alone method of assessing and diagnosing cognitive impairment (Murphy et al., 2020). This is supported by our data, according to which in more than 60% of cases a caregiver made use of the PTT appointment rather than the patient. For patients triaged as yellow or red, the PTT conversation was even conducted exclusively with a caregiver or at least in the presence of a caregiver in 100% of cases. For this reason, an additional appointment with the patient would have been necessary anyway. The sensitivity of PTT with caregivers for detecting cognitive and functional impairment in patients with an urgent need for an initial on-site dementia assessment (yellow and red triage) was nevertheless high. We found significantly higher cognitive and functional impairment, higher BPSD severity, but shorter waiting times in the yellow/red triaged groups compared to the green triaged group. Compared to the data collected before the introduction of PTT, where there were no significant differences in waiting times between the different diagnoses, PTT significantly shortened waiting times for patients diagnosed with dementia at initial on-site assessment.

The introduction of PTT had no impact on demographic variables such as average age, gender distribution, severity of cognitive symptoms, or deficits in activities of daily living at the initial on-site assessment. However, the proportion of people without objectifiable cognitive deficits fell by almost two-thirds. The reduction in the number of comprehensive dementia assessments in cognitively healthy people - and thus in those for whom psychoeducation and psychological counseling regarding subjective cognitive deficits is sufficient - can be considered a success. This assumption is in accordance with previous reports on the benefits of psychoeducation aimed at healthy brain aging for older people at risk for dementia (Norrie et al., 2011). Overall, we hypothesize that PTT counseling may help individuals with subjective cognitive decline and concerns about dementia to reduce psychological distress by providing information on how to actively reduce dementia risk.

With regard to the new mAB therapies for AD, it will be essential to identify patients with a possible indication for these treatment options. As specialized units for the diagnosis of AD with the possibility of necessary biomarker assessment, memory clinics will face an increase in referrals for diagnostic clarification after regional approval. The results of Phase II and Phase III trials with lecanemab and donanemab (Sims et al., 2023; van Dyck et al., 2023) have shown that a significant number of patients who appeared to be eligible for antibody therapy turned out to be screening failures (approximately 60–80%). Within our sample, 45% of the patients would not be eligible for any mAB therapy due to advanced dementia severity at the time of initial assessment. A further 35% would miss eligibility criteria due to severe cerebrovascular pathology, ongoing use of anticoagulants, or other exclusion criteria. The majority of MCI patients would fulfil core eligibility criteria for mAB treatment based on MMSE scores, age, and cerebral imaging criteria (detailed results of mAB eligibility analysis not presented). However, based on the PTT criteria, these patients were mostly classified as “non-acute” and assigned to the green triage group, which was associated with a longer waiting time. This situation raises an ethical dilemma that society and researchers in the field of AD have already grappled with (Cummings et al., 2023). Memory clinics will have to decide whether to focus on the timely assessment of people with memory complaints that may be due to AD in order to provide mAB treatment to as many of them as possible, or to prioritize patients with a high burden due to advanced disease severity, BPSD, lack of care and support, and an urgent need for assistance. In the near future, the increasing number of referrals of people with mild cognitive symptoms and a desire to assess eligibility for amyloid antibody therapy is likely to overburden the healthcare system. PTT could therefore be a way to better utilize local resources based on structured and professional assessment of symptoms, provision of psychological counseling or psychoeducation, and evaluation of the indication for dementia assessment.

## Conclusions

Our results show that the introduction of PTT significantly reduced waiting times for initial assessment at a memory clinic, specifically for patients with dementia with an acute need for timely assessment. We expect that the PTT system will lead to a reduction in emergency admissions, thereby reducing the burden on caregivers and the healthcare system. The telephone psychological counseling saved staff and time resources and provided a useful service for people without a current indication for a dementia assessment. This PTT concept can thus help to better manage the increasing need for initial assessments in the context of new therapies for AD and the increasing prevalence of dementia in general.

## Declaration of competing interest

The authors declare that they have no known competing financial interests or personal relationships that could have appeared to influence the work reported in this paper.

## Appendix Supplementary materials

Image, application 1

Image, application 2
