# Supplementary material for: Psychological telephone triage system for outpatient memory clinics - a way for adaptation to new challenges of increasing dementia prevalence and new treatment options?
Source: Int J Clin Health Psychol. 2024 Dec 6;24(4):100530. doi: 10.1016/j.ijchp.2024.100530 (PMC11665686; doi:10.1016/j.ijchp.2024.100530)
Supplement: Supplementary file 1 [file mmc1.docx]

Supplement 1: Detailed description of the pre-screening protocol and the Psychological Telephone Triage survey documentation

**1. Interdisciplinary pre-screening of medical history**

| **A. Review of information regarding the reason for scheduling an appointment at the memory clinic for initial assessment** (who called, which symptoms were reported, ..) | | | | | | | | | | | |
| --- | --- | --- | --- | --- | --- | --- | --- | --- | --- | --- | --- |
| **B. Pre-screening of patient´s medical history regarding pre-existing or concomitant illnesses, risk factors and current medication** | | | | | | | | | | | |
| **Demographic data:** | | | | | | | | | | | |
| **Name:** | | | **Age:** | | | **Date of birth:** | | | | | **Gender:** |
| **Pre-existing or concomitant illnesses** | | | | | | | | **Risk factors** | | | |
| **Cardiovascular/ internal disease** | | **Cerebrovascular/neurological disease** | | | | | | Hearing loss | | | |
| Coronary artery disease | | Parkinson-disease | | | | | | Visual impairment (glaucoma/cataract) | | | |
| Hypertension | | History of seizures | | | | | | Intellectual disability | | | |
| Diabetes | | Major cerebrovascular disease | | | | | | History of recent delirium | | | |
| Dyslipidemia | | History of stroke/TIA | | | | | | Recent surgery | | | |
| Atrial fibrillation | | History of traumatic brain injury | | | | | | **Psychiatric** | | | |
| History of myocardial infarction | | Restless legs syndrom | | | | | | Affective disorder | | | |
| other | | other | | | | | | Schizophrenia/other psychotic disorder | | | |
| other | | other | | | | | | Substance abuse | | | |
| other | | Dementia: □ AD, □ VD, □ PDD, □ other dementia | | | | | | | | | |
| **Relevant medication** | |  | | | | | |  | | | |
| Anti-dementia drugs | | Anticoagulants | | | | | | Psychotropic drugs | | | |
| other | | | | | | | | | | | |
| **Relevant previous findings** | | | | | | | | | | | |
| Neuroimaging | no | | | yes o MRI o CCT → | | | | | Date: | | |
| Previous report of another memory clinic | no | | | yes | | | Location/Date: | | | | |
| Appointment made at another memory clinic | no | | | yes | | | Location/Date: | | | | |
| History of **recent** hospitalization in neurology or psychiatry | no | | | yes | | | Diagnosis/Date: | | | | |
| Social work documentation available | no | | | yes | | | Care level:___, legal representative:_____________ | | | | |
| **C.** **Interdisciplinary assessment of necessity and priority of initial assessment based on pre-screening information** | | | | | → green or yellow triage likely | | | | | → red triage likely | |
| ↓  No indication (blue triage) likely  **→ preparation of information material for psychological telephone counseling** | | | | | ↓  pre-planning of a non-/subacute appointment according to available resources | | | | | ↓  pre-planning of an acute appointment according to available resources | |

**2. Psychological Telephone Triage (PTT) System**

**Survey documentation**

| **Date of appointment for PTT (secretary’s office): Date PTT:** | | | | | | | | | | |
| --- | --- | --- | --- | --- | --- | --- | --- | --- | --- | --- |
| **Caller**  Patient  Relative  Caregiver  Referring physician  other__________________________ | | | | | | | | | | |
| **Who recommended a dementia evaluation**:  Own desire  Family  Physician (0 General practitioner, 0 Psychiatrist, 0 Neurologist)  other __________________ | | | | | | | | | | |
| **Reason for dementia evaluation**:  Acute cognitive symptoms  BPSD  Financial reasons  Preventive care  Positive family history for dementia  Need for care and support  other__________________________ | | | | | | | | | | |
| Have you/the patient had any **critical life events** recently?  no  yes → 0 death or severe illness of a family member, 0 acute somatic disease, 0 acute psychiatric disease, 0 recent surgery, 0 major psychosocial stress, other________________________________ | | | | | | | | | | |
| **Patient´s marital status** | | | | | | | | | | |
| Single | | Married | | | Divorced/Separated | | | | Widowed | |
| **Patient´s living situation?** | | | | | | | | | | |
| Alone | | At home with a partner | | | At home with family | | | | In a nursing home | |
| **Patient´s current care situation?** | | | | | | | | | | |
| No care | Outpatient care | | 24-hour nursing care | | | | Day care | | | Family care |
| Has the patient already been evaluated for memory impairment? (e.g, in private practice) | | | | | | | | **If yes – when and where?** | | |
| Pre-treatment/current treatment  0 Psychiatrist , 0 Neurologist , 0 Psychotherapist | | | | | | | | **Location/Date:** | | |
| Preliminary findings of neuroimaging? (e.g., in private practice, abroad) | | | | no | | yes → | | o Date MRI:  o Date CCT: | | |
| Known previous diagnosis of dementia? | | | | no | | yes → | | Date: | | |

| **Psychological interview by phone** |  | | |
| --- | --- | --- | --- |
| **Did the patient experience the following symptoms during the last month? (Items based on abbreviated CDR rating)** | **no** | **mild** | **marked** |
| Problems with memory (e.g., recalling recent events or forgetting important information) |  |  |  |
| Misplacing things or objects (e.g., keys or glasses) |  |  |  |
| Speech difficulties (e.g., word-finding difficulties) |  |  |  |
| Concentration problems (e.g., during reading) |  |  |  |
| Orientation problems (time or place) |  |  |  |
| Difficulties in coping with familiar tasks ( e.g., housework, cooking, banking ) |  |  |  |
| Difficulties in operating devices such as the telephone/mobile phone or television |  |  |  |
| Difficulties in taking medication correctly and regularly |  |  |  |
| Has less pleasure in things he/she usually likes to do |  |  |  |
| Loss of interest in hobbies and leisure activities |  |  |  |
| **Requires assistance with the following basic functions** | **no** | **mild** | **marked** |
| Personal hygiene |  |  |  |
| Toilet use |  |  |  |
| Getting dressed |  |  |  |
| Nutrition |  |  |  |
| **Did the patient experience the following behavioral or emotional symptoms during the last month?** | **no** | **mild** | **marked** |
| Sleep disturbances (e.g., difficulties falling asleep or staying asleep) |  |  |  |
| Social withdrawal - avoids social contact |  |  |  |
| Appears sad, hopeless, or depressed |  |  |  |
| Appears aggressive, agitated, or psychotic |  |  |  |
| **Care and support** | **yes** | **partially** | **no** |
| Does the patient have sufficient care and support? |  |  |  |
|  | **↓** | | |
|  | **Triage according to PTT criteria** | | |

Abbreviations: Alzheimers´s disease (AD), Vascular dementia (VD), Parkinson´s disease dementia (PDD), Behavioral and psychological symptoms in dementia (BPSD), Magnetic resonance imaging (MRI), Cerebral computer tomography (CT), Clinical Dementia Rating Scale (CDR)
