# Supplementary material for: Psychological telephone triage system for outpatient memory clinics - a way for adaptation to new challenges of increasing dementia prevalence and new treatment options?
Source: Int J Clin Health Psychol. 2024 Dec 6;24(4):100530. doi: 10.1016/j.ijchp.2024.100530 (PMC11665686; doi:10.1016/j.ijchp.2024.100530)
Supplement: Supplementary file 2 [file mmc2.docx]

Supplement 2. Description of the 4-level Psychological Telephone Triage (PTT) system

| **Triage criteria** | Green - Not acute | Yellow - Subacute | Red - Acute | Blue - No indication | |
| --- | --- | --- | --- | --- | --- |
| Indication | Screening, subjective cognitive decline | Suspected mild dementia | Suspected moderate to severe dementia with BPSD | No indication for initial assessment at the memory clinic due to | |
| Referring person | Self-assignment or recommended by family/caregiver | Evaluation recommended by primary care or specialist | Referral from primary care or specialist | Dementia assessment already undertaken or planned elsewhere | Acute or severe somatic and/or psychiatric disorder that conflicts with dementia assessment |
| Cognitive impairment (CI) and orientation | No or slight CI, fully oriented | Mild to moderate CI interfering with IADL, orientation difficulties | Moderate to severe CI interfering with IADL, Temporal and local orientation impaired |  |  |
| BPSD | Not present or mild non-disruptive and/or psychotic BPSD | Suspected mild BPSD with disruptive and/or psychotic symptoms | Moderate or severe BPSD (disruptive and/or psychotic and/or agitation) | Patient refusal of dementia assessment | Request for advice on memory training or advice for relatives |
| IADL | Not or mildly impaired | Mildly impaired | Severely impaired |  |  |
| Basic functions | Not impaired | Not or mildly impaired | Severely impaired |  |  |
| Risk factors/critical life events | Family history negative or positive for dementia  Absence of or adequately controlled psychiatric and/or somatic comorbidities  Critical life events | Positive family history for dementia  Partially controlled psychiatric and/or somatic comorbidities  Critical life events | Positive family history for dementia  Insufficiently controlled psychiatric and/or somatic comorbidities  Critical life events |  |  |
| Care and support | Available or not necessary | Partially available/possible | Not available or not fully available |  |  |
| Time pressure | None | Mild due to:  - Mild patient and/or caregiver burden  - Care planning  - Financial support | High due to  - Severe patient and/or caregiver burden  - Missing care and support or insufficient financial support  - Urgent need for institutionalization and/or legal representation |  |  |

Behavioral and Psychological Symptoms in Dementia (BPSD), Instrumental Activities of Daily Living (IADL), Subjective cognitive decline (SCD)
